# Feasibility and Acceptability of a Positive Psychological Intervention for Patients With Metastatic Breast Cancer: Pre-Post Pilot Study

**DOI:** 10.2196/77636

**Published:** 2025-10-07

**Authors:** Claire C Conley, Elizabeth L Addington, Mikaela Velazquez-Sosa, Brenna Mossman, Lesley Glenn, Shontè Drakeford, Roxana Guerra, Claudine Isaacs, Ami Chitalia, Christopher Gallagher, Suzanne C O'Neill, Judith T Moskowitz

**Affiliations:** 1 Department of Oncology, Georgetown University, 2115 Wisconsin Ave NW, Suite 300, Washington, DC, 20007, United States, 1 2026875086; 2 Department of Medical Social Sciences, Feinberg School of Medicine, Northwestern University, Chicago, IL, United States; 3 Patient Author, Project Life MBC, Calabasas, CA, United States; 4 Patient Author, Upper Marlboro, MD, United States; 5 Patient Author, Washington, DC, United States; 6 MedStar Washington Hospital Center, Washington, DC, United States

**Keywords:** advanced cancer, depression, anxiety, positive psychology, ecological momentary intervention

## Abstract

**Background:**

Depression and anxiety are prevalent among patients with metastatic breast cancer (MBC), but there are few evidence-based psychological interventions specifically designed for this population.

**Objective:**

This study aimed to assess the feasibility, acceptability, and clinical impact of a multicomponent positive psychological intervention, enhanced with an ecological momentary intervention for symptom management, for patients with MBC.

**Methods:**

We recruited patients with MBC from a National Cancer Institute–designated comprehensive cancer center. Participants completed 5 weekly virtual individual sessions with a study counselor focused on positive emotion regulation skills. Participants also reported physical and psychological symptoms daily between sessions via SMS text messaging. Clinically elevated symptoms triggered a personalized coaching SMS text message tailored to the symptoms reported and the skills learned that week. Primary outcomes were intervention feasibility and acceptability. We also examined pre- to postintervention changes in depression, anxiety, positive affect, and positive emotion regulation skill use. Finally, a subset of participants completed qualitative exit interviews focusing on their experience in the study; interview data were analyzed using rapid qualitative analysis.

**Results:**

We approached 20 patients with MBC, established contact with 15 (75%), received consent from 10 (67%), and retained 9 (90%) patients through the end of the study. Participants were 55 (SD 14.4, range 35-75) years old on average and identified as non-Hispanic White (5/10, 50%), non-Hispanic Black (4/10, 40%), or Latina (1/10, 10%). Participants attended 92% (46/50) of intervention sessions (mean 50, SD 9, range 36‐71 min). On average, they completed 85% (SD 18%, range 46%-100%) of daily symptom assessments and received 23 (SD 5, range 13‐31) coaching messages. Participants reported high perceived intervention feasibility (mean 4.81/5, SD 0.44), acceptability (mean 4.78/5, SD 0.33), and appropriateness for patients with MBC (mean 4.83/5, SD 0.35), above our a priori cutoff of ≥4. All 9 participants (n=9, 100%) recommended the intervention for other patients with MBC. We observed pre- to postintervention decreases in depression (*d*=−0.32) and anxiety (*d*=−0.27) and increases in positive affect (*d*=0.30) and positive emotion regulation skill use (*d*=0.99). Rapid qualitative analysis results demonstrate participants’ positive experiences with the intervention, as well as suggestions for improvement.

**Conclusions:**

This pilot study supports the feasibility of enrolling and retaining racially and ethnically diverse patients with MBC to this trial, the acceptability of the positive psychological intervention enhanced with ecological momentary intervention, and preliminary intervention impacts on depression, anxiety, positive affect, and positive emotion regulation skill use. A large-scale randomized controlled trial is needed to assess intervention efficacy for outcomes of interest.

## Introduction

Metastatic breast cancer (MBC) is breast cancer that has spread to other parts of the body; it is typically considered to be treatable, but incurable [1]. In the past decade, novel treatment approaches have resulted in significantly increased survival times following MBC diagnosis [2-4]. For this reason, the MBC population is anticipated to increase in the coming years; by 2030, an estimated 246,000 people in the United States will be living with MBC (a 59% increase from 2017) [5,6].

Living with MBC can affect psychological functioning. An estimated 20% to 52% of patients with MBC report clinically significant depression, and 36% to 60% report clinically significant anxiety [7-12]. However, most interventions to improve depression and anxiety in patients with breast cancer are designed for those with early-stage disease. The needs of patients with MBC are different from those with early-stage breast cancer [13], and resources that are not specific to MBC do not adequately meet the needs of this population [14]. Given their unique experiences, interventions addressing depression and anxiety, specifically for patients with MBC, are urgently needed.

There are few evidence-based interventions targeting depression and anxiety for patients with MBC. A 2023 systematic review identified 13 studies testing supportive care interventions designed to improve quality of life and symptom experience among patients with MBC [15]. Of the 7 studies that examined depression or anxiety as an outcome, only 2 demonstrated statistically significant improvement in these symptoms [16,17]. Thus, further research is needed to optimize interventions targeting depression and anxiety specifically for patients with MBC through the integration of novel intervention targets and methods.

Incorporating a focus on positive affect may be one way of optimizing psychological interventions for patients with MBC. Positive affect refers to pleasant feelings and moods, such as peace and joy [18]. Positive affect is *not* the same as absence of negative affect, as individuals can experience both positive and negative affect at the same time, and a high level of one does not necessitate a low level of the other [19,20]. Our prior work highlights the importance of positive affect among those with MBC and demonstrated that positive affect significantly predicted quality of life, above and beyond the effects of sociodemographic characteristics, clinical characteristics, and physical symptoms [21].

Positive psychological interventions (PPIs) are a promising approach to reduce depression and anxiety *and* increase positive affect among patients with MBC. PPIs focus on building practical skills designed to improve well-being [22,23]. Some PPIs focus on a single skill (eg, gratitude), while others teach several skills. In a prior pilot of a PPI among those with MBC, we demonstrated a reduction in depression but no improvement in positive affect [24]. Thus, prior pilot data suggested a need for further adaptations to the PPI to boost intervention effects on positive affect.

One potential explanation for these findings is the lack of PPI skill use during moments of higher negative affect when they are most needed. Nudge Theory suggests that behavior is susceptible to contextual factors (ie, how information is presented or structured) [25-28]. Thus, using nudges to influence choices is a potential strategy for behavior change [29]. Responsive behavioral nudges, triggered by participants’ own symptom thresholds, engage patients when they are most receptive to help [30]. Compared to nudges that are not responsive (ie, daily reminders), responsive nudges can prevent habituation (ie, decreased response to an intervention over repeated exposures) [31]. Responsive interventions have been shown to improve positive affect in other populations and settings [32-34]. Thus, the previously observed null effects of the PPI on positive affect among patients with MBC might be addressed through responsive behavioral nudges.

In adapting the PPI in line with Nudge Theory, we incorporated an ecological momentary intervention (EMI). EMI delivers dynamic, individually tailored treatments to patients in real time and in natural settings [31,35]. EMI has several benefits over traditional “one-size-fits-all” interventions: maintaining participant engagement, sustaining continued behavior change for longer durations, and ultimately achieving greater intervention effects [36]. However, EMI has rarely been used in cancer survivorship research [37]. To our knowledge, there are no studies examining EMI in the context of advanced cancer.

To address these research gaps, we conducted a single-arm pilot study examining the feasibility and acceptability of a multicomponent PPI—enhanced with an EMI for symptom management—for patients with MBC. The aims of this study were 2-fold: (1) to demonstrate the feasibility and acceptability of the intervention for patients with MBC and (2) to examine pre- to postintervention changes in depression, anxiety, positive affect, and positive emotion regulation skill use.

## Methods

### Study Design

Following best practices for the development of behavioral interventions [38], we conducted a single-arm pilot study (see Checklist 1 for CONSORT [Consolidated Standards of Reporting Trials] checklist for reporting a pilot or feasibility trial [39]). Although this design does not allow us to assess intervention efficacy, we are able to assess intervention feasibility, acceptability, and clinical impact on outcomes of interest—the primary goals of this project. This research was designed and conducted with the active input of patient advocate collaborators to ensure the methodology was informed by the perspectives of individuals living with MBC. Qualitative data collection and analyses align with the COREQ (Consolidated Criteria for Reporting Qualitative Research; see Checklist 2) [40].

### Participants and Procedures

Eligible participants were those diagnosed with MBC, assigned female at birth, aged ≥18 years, and able to communicate verbally in English. Participants were also required to have a working telephone capable of receiving SMS text messages and accessing the internet to facilitate engagement with the EMI. We excluded individuals with a life expectancy of <6 months as assessed by their treating breast oncologist before study enrollment [41].

The target sample size for this feasibility study was 10 participants and was determined based on recommendations for pilot and feasibility studies in the palliative care setting [42]. Participants were recruited from a previous study cohort. Between January 2023 and April 2024, we conducted an observational study of quality of life among patients being treated for MBC at 5 medical oncology clinics in the mid-Atlantic region (N=125) [21]. A subset of participants (96/125, 77%) had agreed to be contacted for future studies. From this group, we selected individuals who had reported elevated levels of depression or anxiety (32/96, 33%), defined as a rating of 7 or higher on a 0 to 10 visual analog scale at any point throughout the study [43]. A member of the research team confirmed patients’ vital status and eligibility with their treating oncologist; 27 (27/32, 84%) patients were alive, and we received oncologist permission to contact 22 (22/27, 81%) patients. Oncologists asked us not to contact 5 patients who had transferred care outside our health care system and were now under the care of another oncologist. Recruitment stopped upon achieving our target sample size of 10 consented participants.

From October to November 2024, a member of the research team contacted potential participants via telephone, introduced the study, and confirmed patient eligibility and interest. Following informed consent, participants completed a baseline (T0) survey assessing sociodemographic characteristics, depression, anxiety, positive affect, and positive emotion regulation skill use. On completion of the baseline survey, participants were scheduled for intervention sessions (see Intervention section below). Immediately post intervention (ie, after the fifth and final intervention session), participants completed a follow-up (T1) survey assessing primary and secondary outcomes (see Measures section below). Follow-up assessments took place between November and December 2024.

Finally, all participants were given the opportunity to complete an optional exit interview. A female research assistant with prior training and experience in qualitative interviewing (MVS) conducted the semistructured exit interviews. The interviewer did not have a relationship with the interviewees before the exit interview. At the start of the interview, the interviewer introduced herself and reminded participants of the purpose for the exit interview (ie, to obtain feedback on the PPI). In terms of positionality [44,45], the interviewer had foundational knowledge of MBC but not firsthand experience. She was also younger than the interview participants and from a different geographic region of the United States. These characteristics may introduce bias by affecting the interviewer’s application of probing questions. Potential bias was managed through postinterview debriefing with the principal investigator (CCC).

Semistructured qualitative interviews explored participants’ experiences with the intervention and suggestions for improvement (see Multimedia Appendix 1 for the interview guide). Interviewees were instructed to find a quiet, private location, without nonparticipants, for the duration of the interview. Interviews were conducted via telephone or video call (as preferred by the participant) and lasted 31 (SD 12, range 13-50) minutes on average. Interviews were audio recorded and transcribed verbatim. The interviewer took field notes during the interviews. As participants did not consent to be recontacted, we did not conduct repeat interviews, return transcripts to participants for review, or have participants provide feedback on the findings.

### Intervention

Positive Emotion Skills Training (POET) is a PPI designed to bolster participants’ ability to cope with major life stressors by increasing their experiences of positive affect [46]. Participants learned 8 evidence-based skills: noticing positive events, capitalizing (ie, maximizing positive experiences), gratitude, mindfulness, positive reappraisal, identifying personal strengths, creating attainable goals, and altruism and acts of kindness. POET includes 5 weekly individual sessions with a facilitator. In this study, sessions took place virtually (ie, via phone, 27/46, 59%, or video conference, 19/46, 41%, as preferred by the participant). Sessions lasted 50 (SD 9, range 36-71) minutes on average. Each session included (1) a brief check-in, (2) home practice review, (3) didactic portion in which the facilitator introduces the skills for that session, (4) interactive practice of the skills, and (5) discussion of home practice for the next week. Didactic content and home practice worksheets were provided as a paper intervention booklet; participants were encouraged to follow along with written content during the session and to document their home practice by writing in the booklet between sessions.

Participants also completed daily symptom reporting between POET sessions. Specifically, participants reported psychological symptoms (eg, depression, anxiety), physical symptoms (eg, pain, fatigue, gastrointestinal distress, nausea, reduced appetite, reduced libido), and positive experiences (eg, social connectedness, peace, joy) on a visual analog scale ranging from 0 to 10. Daily symptom reporting surveys were delivered via SMS text messages once per day, between 5 PM and 11 PM; to maximize the likelihood of survey completion, the exact time of survey delivery was individualized and based on participant preference.

Our intervention also incorporated an EMI, which was based on participants’ responses to the daily symptom monitoring surveys. Specifically, high levels of symptoms on daily symptom monitoring surveys (scores≥7) triggered a next-day coaching message delivered by SMS text messages. Coaching messages were developed by adapting language from the existing intervention worksheets. Our multidisciplinary study team and patient advocates also provided input on the coaching messages to ensure their relevance to individuals living with MBC. The goal of the coaching messages was to encourage the use of positive emotion regulation skill(s) learned in POET sessions. Thus, coaching messages were specific to both the symptom(s) reported and the skill(s) that the participant learned in their most recent POET session. The coaching message was delivered the morning following the participant’s evening symptom reporting survey, at approximately 10 AM.

### Measures

#### Feasibility and Acceptability

At T1, participants completed the Feasibility of Intervention Measure, Acceptability of Intervention Measure, and Intervention Appropriateness Measure [47]. Each measure includes 4 items, which are rated on a 5-point Likert scale, ranging from “completely disagree” (1) to “completely agree” (5) and averaged to create scale scores ranging from 1 to 5 (higher=greater). Scores ≥4 indicate adequate levels of intervention feasibility, acceptability, and appropriateness.

In addition, participants provided feedback on the number of sessions in the POET intervention (too few, too many, or just right), the length of each POET session (too short, too long, or just right), preferred format for the POET intervention (eg, individual, group, self-guided website, in-person, virtual), and whether they would recommend the POET intervention for other people with MBC (yes or no).

#### Depression

Participants completed the Patient-Reported Outcomes Measurement Information System (PROMIS) Depression 4-item short form at T0 and T1 [48,49]. Items are rated on a 5-point Likert scale, ranging from “never” (1) to “always” (5), summed, and converted to T-scores, which enable comparison to the general population (mean 50*,* SD 10).

#### Anxiety

Participants completed the PROMIS Anxiety 4-item short form at T0 and T1 [48,50]. Items are rated on a 5-point Likert scale, ranging from “never” (1) to “always” (5), summed, and converted to T-scores, which enable comparison to the general population (mean 50, SD 10).

#### Positive Affect

Participants completed the positive affect subscale of the Modified Differential Emotions Scale (mDES) at T0 and T1 [51]. In total, 10 items are rated on a 5-point Likert scale, ranging from “never” (0) to “most of the time” (4), indicating the extent to which emotions have been experienced within the last 24 hours. Items are averaged to create a subscale score ranging from 0 to 4 (higher=more).

#### Positive Emotion Regulation Skill Use

As in prior work, we assessed participants’ use of the positive emotion regulation skills taught in the POET intervention in the last week [24]. Participants respond to 11 items on a 5-point Likert scale, ranging from “definitely false” (1) to “definitely true” (5). Item scores are averaged to create a total score ranging from 1 to 5 (higher =greater).

#### Demographic Characteristics

At T0, participants self-reported their age, sex assigned at birth, gender identity, sexual orientation, race, ethnicity, education, employment status, annual household income, and insurance status.

#### Clinical Characteristics

Time since MBC diagnosis and disease subtype were abstracted from the electronic medical record.

### Statistical Analysis

#### Quantitative

Descriptive statistics were used to characterize the sample and to examine measures of intervention feasibility, acceptability, and appropriateness. Student *t* tests were used to examine changes in depression, anxiety, positive affect, and positive emotion regulation skill use from T0 to T1. Analyses were conducted in SPSS (version 28; IBM Corp). All tests were 2-tailed, and significance was specified as *P*<.05. Cases with missing outcome data were deleted listwise.

#### Qualitative

Qualitative analysis was guided by a descriptive phenomenological approach, wherein the researchers sought to understand the unique lived experience of individuals by exploring the meaning of a phenomenon [52]. Exit interview transcripts were analyzed by 2 members of the study team (CCC, MVS) through a rapid qualitative analysis (RQA) procedure [53]. Main topics (“domains”) were drawn from the interview guide, and a summary template was developed (see Multimedia Appendix 2 for the RQA template). Team members used the template to summarize interview transcripts. Summary templates were compiled into a single matrix reflecting the depth and breadth of information for each domain [54]. Although our sample size for qualitative exit interviews was limited to participants in the pilot intervention, we did obtain data saturation with 9 completed exit interviews [55].

### Ethical Considerations

All study procedures were reviewed and approved by the institutional review board at Georgetown University (IRB #00008193). Study procedures ensured that participants’ privacy and confidentiality were maintained. Patients provided written informed consent via an electronic informed consent form. Participants received a US $25 gift card on completion of each survey. Participants received a US $50 gift card on interview completion.

## Results

### Descriptive and Preliminary Analyses

**Figure 1.**
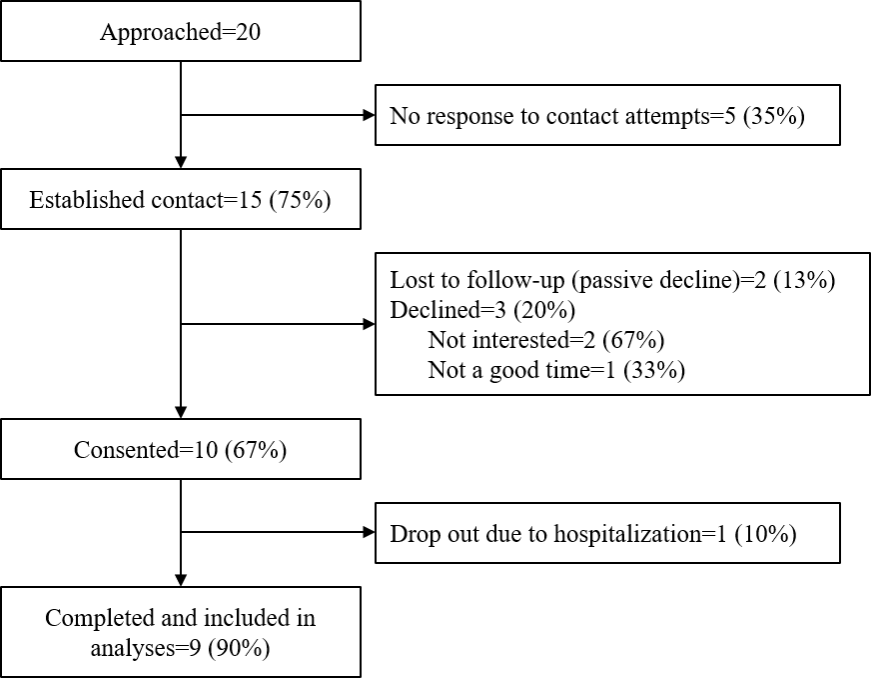
Study flowchart.

We approached 20 patients with MBC and established contact with 15 (15/20, 75%) patients; 10 (10/15, 67%) patients provided consent (Figure 1). One participant withdrew due to prolonged hospitalization, and 9 (9/10, 90%) were retained through the study.

Table 1 provides a full description of the sample. All participants self-identified as cisgender heterosexual women. The average participant age was 55 (SD 14, range 35‐85) years. The 10 participants identified as non-Hispanic White (n=5, 50%), non-Hispanic Black (n=4, 40%), or Latina (n=1, 10%). Most of the participants had a college degree or greater (n=9, 90%), were not working (n=5, 50%), had an annual household income greater than US $100,000 (n=5, 50%), and had private health insurance (n=9, 90%). The median time since MBC diagnosis was 3.7 years, but this ranged widely from a minimum of 1.5 years to a maximum of 18.1 years. Most participants (n=7, 70%) had hormone receptor–positive, human epidermal growth factor receptor 2–negative breast cancer.

**Table 1.** Participant demographic and clinical characteristics (N=10).

Characteristics	Values
Demographic characteristics	
Age (years), mean (SD)	55.2 (14.4)
Race and ethnicity, n (%)	
Non-Hispanic White	5 (50)
Non-Hispanic Black	4 (40)
Latina	1 (10)
Education, n (%)	
High school degree or equivalent	1 (10)
College degree	6 (60)
Postgraduate degree	3 (30)
Employment status, n (%)	
Working full time	1 (20)
Working part time	3 (30)
Retired	4 (40)
Unable to work	1 (10)
Annual household income, n (%)	
<US $100,000	4 (40)
≥US $100,000	5 (50)
Prefer not to answer	1 (10)
Health insurance type^a^*,* n (%)	
Private	8 (80)
Public (eg, Medicare, Medicaid)	4 (40)
Military or veterans health care	1 (10)
Clinical characteristics	
Years since MBC^b^ diagnosis, median (range)	3.7 (1.5‐18.1)
Breast cancer subtype, n (%)	
HR^c^ positive and HER2^d^ positive	2 (20)
HR positive and HER2 negative	7 (70)
Triple negative	1 (10)

a As participants could select more than one, does not sum to 100%.

b MBC: metastatic breast cancer.

c HR: hormone receptor.

d HER2: Human Epidermal Growth Factor Receptor 2.

At baseline, participants demonstrated more depression (mean 55.4, SD 8.2) and anxiety (mean 57.1, SD 9.0) than the general population (mean 50*.*0, SD 10). These scores are indicative of mild depression and anxiety [56].

### Feasibility and Acceptability

Participants attended 92% (46/50) of intervention sessions; specifically, 9 participants attended all 5 intervention sessions, and 1 participant attended only 1 intervention session before dropping out. Intervention sessions lasted 50 (SD 9, range 36-71) minutes on average.

For participants who were retained for the duration of the intervention, the average length of the intervention was 29 (SD 2, range 28‐34) days. A total of 8 participants completed the 5 weekly intervention sessions in exactly 5 weeks, and 1 participant took an additional week after rescheduling due to competing demands. As symptom assessments were completed daily over the course of the intervention period, the possible number of daily symptom assessments ranged from 28 to 34, depending on the participant. Completion of daily symptom assessments ranged widely, from 46% (13/28) to 100% (28/28). On average, participants completed 85% (SD 18%) of daily symptom assessments. The EMI was triggered for 93% (204/220) of daily symptom assessments. Participants received an average of 23 (SD 5, range 13‐31) coaching messages, corresponding to 4‐5 coaching messages per week in the intervention.

Participants reported high perceived intervention feasibility (mean 4.81, SD 0.44), acceptability (mean 4.78, SD 0.33), and appropriateness for patients with MBC (mean 4.83, SD 0.35). The average scores on all measures were above our a priori cutoff of ≥4, indicating adequate intervention feasibility, acceptability, and appropriateness. No harms or unintended intervention effects were identified.

Most of the 9 participants (n=6, 67%) felt that the number of POET sessions was “just right,” while the remainder (n=3, 33%) felt that there were “too few” sessions. The length of the sessions was also rated by most as “just right” (n=7, 78%), with a few participants feeling the sessions were “too short” (n=1, 11%) or “too long” (n=1, 11%). In terms of intervention format, most participants (n=8, 89%) preferred individual meetings with the study counselor, either via telephone (n=5, 56%) or video call (n=4, 44%). Fewer participants (n=2, 22%) were interested in an in-person intervention. All participants (n=9, 100%) recommended the intervention for other patients with MBC.

### Depression, Anxiety, Positive Affect, and Positive Emotion Regulation Skill Use

The average time between T0 and T1 was 43.6 (SD 8.7) days. Although there were no statistically significant changes in depression, anxiety, or positive affect from T0 to T1 (*P* values ≥.37; Table 2), we did observe small-to-moderate pre- to postintervention decreases in depression (*d*=−0.32) and anxiety (*d*=−0.27) and increases in positive affect (*d*=0.30). This corresponded to clinically significant reductions in depression and anxiety for 55% (5/9) and 33% (3/9) of participants, respectively [57]. We also observed a statistically significant increase in positive emotion regulation skill use (*t*_8_=2.95; *P*=.02), corresponding to a large effect size (*d*=0.99).

**Table 2.** Pre- to postintervention changes in depression, anxiety, positive affect, and positive emotion regulation skill use (N=9).

Construct	T_0_, mean (SD)	T_1_, mean (SD)	*t* test (*df*)	*P* value	*d*	95% CI
PROMIS^a^ depression	55.33 (8.73)	51.99 (7.86)	–0.95 (8)	.37	–0.32	–0.98 to 0.37
PROMIS anxiety	57.23 (9.53)	53.90 (8.48)	–0.81 (8)	.44	–0.27	–0.93 to 0.40
mDES^b^ positive affect	2.04 (1.26)	2.31 (1.06)	0.91 (8)	.39	0.30	–0.38 to 0.97
Positive emotion regulation skill use	3.92 (0.86)	4.28 (0.67)	2.95 (8)	.02	0.99	0.16 to 1.77

a PROMIS: Patient-Reported Outcomes Measurement Information System.

b mDES: Modified Differential Emotions Scale.

### RQA Results

Table 3 includes representative quotes from RQA, highlighting the feasibility, acceptability, and appropriateness of the intervention for people with MBC. All interviewees had positive experiences in the study, describing the POET program as useful, meaningful, or therapeutic. The weekly sessions helped many participants feel engaged or accountable; some participants expressed interest in adding additional sessions or having sessions every other week (to allow more time to practice POET skills). Interviewees also provided suggestions for improving the daily symptom reporting, including adding more emotions and open-ended questions to provide clarifications on their answers.

**Table 3.** Representative quotes from rapid qualitative analysis highlighting intervention feasibility, acceptability, and appropriateness.

Participant	Illustrative quote
No. 4, living with MBC^a^ for 4 years	“People use [these skills] every day. They just don’t know how to categorize them in a way you put on paper.”
No. 5, living with MBC for 1.5 years	“And I loved the program, to be honest, I felt like it’s a lot of stuff that a lot of people need... especially with cancer, you can become, ‘Woe is me,’ kind of quick. And so, it’s a good way to keep it centered on the good in your life. And it’s okay to have those bad days. But to have something to bring you back, I liked that a lot.”
No. 7, living with MBC for 1.5 years	“If [a patient with MBC] were on the fence, that they should pursue the program, I would also say just go into it with an open mind... that the course content, that is actually beneficial.”
No. 8, living with MBC for 3 years	“Mindfulness is one of those skills that helped me stop and realize that it’s okay to put myself into the equation as well and focus on my own, which is something I often tend to ignore.”
No. 9, living with MBC for 18 years	“I’m grateful for the opportunity that someone kind of feels like maybe we do need something different than the pink ribbon.”
No. 10, living with MBC for 3 years	“[The coaching message] was cool. It cheered you up, like, ‘Oh, you did this. Now, why don’t you focus on this?’ And it gives you a guide during the day on what to do instead of just being around your daily life and events and house chores and stuff like that. So that was good.”

a MBC: metastatic breast cancer.

Most interviewees felt that all the skills covered in the POET program were helpful, applicable, or important. However, different participants preferred different skills; several individuals expressed interest in a tailored program, where they could “pick and choose” the skills that felt most important and relevant to them. Relatedly, many participants noted that they were already familiar with and practiced some of these skills. For some, the POET program was a helpful reminder of their skills, while others felt it was redundant to cover this material.

Interviewees generally liked the EMI component of the intervention and appreciated the structure that the coaching messages provided, reminding them to use the skills that they had learned. Some interviewees also reflected that it helped them to stay engaged in the POET program, rather than going a whole week between contacts. Interviewees felt that the frequency of the coaching messages was appropriate. However, 1 participant suggested that it would be nice to receive the coaching message daily, even if they did not complete the daily symptom assessment the day prior.

Finally, interviewees were asked about alternative delivery methods and formats for the POET program. While most participants were interested in the option of a self-paced web-based program, they still wanted to have weekly meetings with a facilitator. One participant suggested a “buddy program” between participants through the web-based platform. Regarding group sessions, interviewees often expressed interest with caution because of concerns about privacy and confidentiality. One participant noted that it would be important for group sessions to be structured and “different from a support group.”

## Discussion

### Principal Findings

In this single-arm pilot study, we demonstrated that a multicomponent PPI incorporating EMI is feasible and acceptable among patients with MBC. Pilot study participants rated the POET intervention as highly feasible, acceptable, and appropriate for patients with MBC. High rates of recruitment and retention also demonstrate the feasibility of this trial. Preliminary data also suggest that the POET intervention may impact key outcomes of depression, anxiety, positive affect, and positive emotion regulation skill use. However, these findings are limited by the study design, including the small sample and lack of a control group, and must be replicated in a fully powered, randomized trial. Altogether, the results of this pilot study provide support for further examination of PPIs in the context of MBC.

Meta-analytic data demonstrate that PPIs have robust effects on the key MBC outcomes identified in our prior work: depression, anxiety, and positive affect [58]. However, in the oncology setting, PPIs have primarily been tested among patients with early-stage cancer [59-61]. A meta-analysis conducted by Casellas-Grau et al [59] identified 16 studies examining PPIs for patients with breast cancer, which they grouped into 5 categories: mindfulness-based approaches, expression of positive emotions, spiritual interventions, hope therapy, and meaning-making interventions. More than half of the included studies recruited only individuals with early-stage cancer; only 1 study focused on MBC specifically [62]. While additional trials have been published since this review was completed, to our knowledge, the initial pilot of POET is the only other study testing a PPI specifically for patients with MBC [24]. This study thus extends prior research in 2 key ways. First, we examine the feasibility and acceptability of a PPI among women with MBC, who have been excluded from previous studies in this space. Second, our PPI was multicomponent and touched on several of the categories identified in the meta-analysis, including mindfulness, expression of positive emotions, hope, and meaning-making. By integrating multiple positive psychological skills, POET may be more appealing to potential participants and could have more robust effects on our outcomes of interest than single-component PPIs.

Results also highlight the potential added benefit of EMI on positive affect for patients with MBC. Despite specifically being designed to increase positive affect, an earlier version of the POET intervention did not demonstrate effects on positive affect among patients with MBC [24], indicating the need for adaptations. Although this small pilot study cannot provide evidence of intervention efficacy, we did observe pre- to postintervention increases in positive affect, possibly due to our consideration of Nudge Theory and the resulting inclusion of EMI. Nudges such as those included in this study can interrupt unwanted behaviors or experiences and prompt positive behavior change. For example, nudge-based interventions have successfully been used to disrupt sedentary time [63,64] and social media use [65]. In the context of PPIs, nudges represent an opportunity to interrupt negative emotions [66], prompt use of positive emotion skills [67], and reduce emotional spillover (when negative emotions experienced in one period continue into a later period) [68-70]. Thus, the responsive behavioral nudges that we incorporated in this intervention may have boosted the effects of POET on positive affect through these mechanisms.

Notably, Salsman et al [71] conducted a meta-analysis examining intervention effects on positive affect among cancer survivors and identified only 3 studies focusing on advanced cancer. Disappointingly, intervention effects on positive affect were stronger when trials focused on individuals with early-stage cancer (average effect size: *g*=0.42), compared to trials enrolling patients with advanced cancer (average effect size: *g*=−0.03). Salsman et al [71] noted that this may be due to differences in affective activation between individuals with early-stage and advanced disease. In other words, patients with advanced disease may tend to experience “low activation” positive affect (ie, peace, tranquility, and contentment), while individuals with early-stage disease may tend to experience “high activation” (ie, excitement). Our measure of positive affect (the mDES) included both low-activation and high-activation emotions. Although this small pilot study was not powered to examine changes in individual mDES items, future research should examine the impact of POET on low- versus high-activation emotions among women with MBC.

The results of this small pilot study also indicate the potential for the POET intervention to improve anxiety among patients with MBC. This is consistent with meta-analytic data demonstrating that PPIs ameliorate anxiety among medically ill patients [72]. However, specifically among people with cancer, 1 meta-analysis demonstrated high heterogeneity across studies in PPIs’ effects on anxiety, potentially resulting in less reliable effect size estimates [73]. The observed high heterogeneity in study findings could be due to differences in study populations, interventions, or outcome measurements. Thus, attention to these details through a rigorous, systematic program of research is needed to generate reliable estimates of the impact of PPIs on anxiety among women with MBC.

The generalizability of these findings is limited by the sociodemographic and clinical characteristics of the sample. While we were able to achieve diversity in terms of participant race and ethnicity, nearly all participants in this pilot study were highly educated and of high socioeconomic status. Prior literature has suggested that socioeconomic factors, such as education level, can moderate the effects of PPIs, particularly for behavioral outcomes (eg, antidepressant use) [74]. Future studies including larger, more socioeconomically diverse samples are needed to support the generalizability of these findings to the larger population of women with MBC. Recruiting outside of National Cancer Institute–designated comprehensive cancer centers may be one strategy to increase participant diversity, as patients presenting for care at these institutions are more likely to be non-Hispanic White, privately insured, and with high socioeconomic status [75]. Furthermore, all participants in this pilot study were able to communicate verbally in English. Prior research suggests that individuals with limited English proficiency have more barriers in accessing cancer care [76] and worse breast cancer outcomes than their English-speaking counterparts [77]. For example, Spanish-speaking Latina immigrant breast cancer survivors experience worse quality of life and emotional well-being than English-speaking survivors [78]. Expanding this PPI beyond English-speaking participants is a necessary next step to improve equity in psycho-oncology research. In terms of clinical characteristics, this study included only participants who reported elevated depression or anxiety in a prior study. While elevated depression and anxiety are common among patients with MBC [7-12], the present sample may not represent the broader MBC population, including those without elevated distress. It is possible that the POET intervention would be less feasible, acceptable, and impactful among patients with MBC and without elevated depression or anxiety.

 Qualitative feedback from intervention participants can be used to guide future iterations of the POET program. Specifically, based on participant feedback, we will revise the daily symptom reporting procedure to include more emotions and open-ended questions. In addition, we are exploring the possibility of tailoring the POET program to individual participants based on their current affective experiences, prior experience with POET skills, or current use of the POET skills. Tailored or adaptive interventions that meet the specific needs and preferences of individual patients may be more acceptable, ultimately increasing participants’ engagement with and benefit from the intervention [31,79]. However, additional research would be needed to identify the decision points at which the POET intervention is tailored and test the effectiveness of the tailored intervention versus standard intervention delivery [80].

### Strengths and Limitations

This pilot study has several strengths. Patients with metastatic cancer have unique psychosocial needs [13,14], yet are underrepresented in research [81,82]. Furthermore, this is among the first studies examining a PPI for patients with MBC; despite the importance of positive experiences for this patient population, prior intervention trials do not focus on increasing positive affect [15,83,84]. Our incorporation of EMI is also a strength, as it may boost intervention results and increase engagement. We were able to recruit and retain a racially and ethnically diverse group of study participants, supporting the feasibility of doing so in a future, larger trial. In addition, participants had been living with MBC for varying lengths of time, providing unique perspectives on the disease and related psychosocial needs. We used robust and well-validated outcome measures with established cutoffs for clinically significant change, enabling us to explore the clinical impact of the POET intervention. Finally, the study was designed in collaboration with patient advocates, ensuring relevance to patients’ lived experiences.

Nonetheless, the results must be interpreted in light of limitations. Most notably, this was a small, single-arm pilot study. While this design is appropriate for our primary goal of assessing intervention feasibility and acceptability [38], we are unable to determine whether improvements in our outcomes of interest are due to the intervention or other factors. A fully powered randomized clinical trial with long-term follow-up is needed to understand the effect of the POET intervention on depression, anxiety, positive affect, and positive emotion regulation skill use among patients with MBC. Moreover, from these pilot findings, we are unable to disentangle the relative effects of the POET sessions and the integrated EMI. A future dismantling study or factorial study would be needed to understand the individual and combined effects of different intervention components [85]. Our outcome measures were self-reported, which may introduce bias due to demand characteristics [86]. Furthermore, in an effort to minimize participant burden, we elected to use very brief measures of depression and anxiety; while they have strong psychometric properties, they may be less sensitive to change than longer versions [87]. Future studies might incorporate longer self-report measures or objective measures to further triangulate intervention effects. Participants in this pilot study were recruited from a prior trial, which included remote symptom reporting via ecological momentary assessment and required participants to own a phone capable of receiving SMS text messages and accessing the internet [21]. This may have resulted in a more “tech-savvy” and less representative sample, and limits generalizability. Only individuals with elevated depression or anxiety were selected, further narrowing the sample and limiting applicability to the broader MBC population. Furthermore, we only contacted individuals who had previously agreed to be contacted for future studies, which may have introduced self-selection bias (ie, individuals who agreed to be contacted for future studies may be more willing to participate). Recruitment rates might be lower among individuals who had not previously expressed interest in participating in research. Most participants in this study were highly educated and of high socioeconomic status, and all were able to communicate verbally in English and were receiving care at a National Cancer Institute–designated comprehensive cancer center. Thus, results may not generalize to the broader, socioeconomically and linguistically diverse population of people with MBC and those receiving care in community oncology settings [88,89]. Finally, our sample was relatively homogenous in terms of MBC subtype, with 70% having hormone receptor–positive Human Epidermal Growth Factor Receptor 2–negative disease. This has significant implications for participants’ lived experience of MBC, including treatment regimen (ie, hormone therapy rather than chemotherapy), time toxicity, and quality of life. Relatedly, none of our participants were within a year of their MBC diagnosis and may therefore be in a different stage of adjustment to their disease. The effect of PPIs may vary for individuals closer to diagnosis and undergoing different types of treatments for MBC. Future studies with more clinically diverse samples are needed to understand if the intervention is more or less impactful for certain cohorts.

### Conclusions

This pilot study demonstrates the promise of the POET program for patients with MBC. Specifically, the results support the feasibility of enrolling and retaining racially and ethnically diverse patients with MBC to this trial, the acceptability of the PPI enhanced with EMI, and potential intervention impacts on depression, anxiety, positive affect, and positive emotion regulation skill use. A large-scale randomized controlled trial is needed to assess intervention efficacy for outcomes of interest.

## Supplementary material

10.2196/77636 Multimedia Appendix 1 Exit interview guide.

10.2196/77636 Multimedia Appendix 2 Rapid qualitative analysis transcript summary template.

10.2196/77636 Checklist 1 CONSORT checklist.

10.2196/77636 Checklist 2 COREQ checklist.

## Abbreviations

CONSORT: Consolidated Standards of Reporting Trials
COREQ: Consolidated Criteria for Reporting Qualitative Research
EMI: ecological momentary intervention
MBC: metastatic breast cancer
mDES: Modified Differential Emotions Scale
POET: Positive Emotion Skills Training
PPI: positive psychological intervention
PROMIS: Patient-Reported Outcomes Measurement Information System
RQA: rapid qualitative analysis
