# Supplementary material for: Feasibility and Acceptability of a Positive Psychological Intervention for Patients With Metastatic Breast Cancer: Pre-Post Pilot Study
Source: JMIR Form Res. 2025 Oct 7;9:e77636. doi: 10.2196/77636 (PMC12504039; doi:10.2196/77636)
Supplement: Multimedia Appendix 1 [file formative-v9-e77636-s001.pdf]

Exit Interview Guide

[TOPIC: Introduction]
Thank you for participating in our study! In this interview, I'll be asking you to tell me about your experience participating in the POET program and your feedback on how we can improve the POET program.

[TOPIC: Experiences in the study]
First, I'd like to ask what it was like for you overall to participate in the POET program. How would you describe your experience?

Potential areas to probe:
- What initially interested you in the POET program?
- What did you like and/or dislike about the POET program?
- How did you feel about the home practice activities that were included in the workbook? Were you able to complete them? If not, what got in the way?
    - How would you feel about doing those activities digitally, instead of on paper?
- How easy was it for you to answer the daily diaries that were sent to you via text message? Did you have any technical difficulties?
- How did you feel about the text messages that you received after completing the daily diaries? Did you like or dislike them?
- Do you have any feedback for us on how we could improve the POET program?

[TOPIC: Intervention schedule]
Second, I'd like to ask for your thoughts about the schedule for the POET program. In this study, we had 5 weekly sessions. How did you feel about this schedule?

Potential areas to probe:
- Were you able to fit the program into your life/your schedule?
- Do you think the program should have more sessions? Fewer?
- Do you think the sessions should be spread out over more time? Should they take place every other week? Monthly?

[TOPIC: Skills introduced in POET program]
Third, I'd like to ask about the specific skills that were discussed in the POET program. You learned about nine different skills, including noticing positive events, capitalizing on positive events, gratitude, mindfulness (formal and informal), positive reappraisal, identifying personal strengths, setting goals, and acts of kindness. What did you think of these skills?

Potential areas to probe:
- Which skills did you like/dislike the most? Why?
- Are there any that you would recommend removing from the POET program?
- Are there any skills that we didn't include that you think would be good for us to add to the POET program?

[TOPIC: Interest in alternative study designs]
For future studies, we're considering taking a different approach to the POET program. I'd like to ask about your interest in different formats for the program. For each one, just let me know what your initial reaction is to that idea…
- Would you be interested in participating in POET in a group format? What if the group took place in-person? What if it took place on Zoom?
- POET has also been delivered as a self-guided program, where you log in to a website each week to learn new content. Would you be interested in participating in a program like that?
- What if we did a combination of the two, where you used the website to learn new material, but then discussed it in a group setting?

[TOPIC: Additional input]
Thinking about your whole experience participating, is there any other feedback you would like to provide?

Potential areas to probe:
- If another person with MBC was considering participating in the POET program, is there any advice you would have for them?
- Is there anything you would change about the POET program?
- Anything else you'd like us to know that would help us to improve the POET program in the future?

[End of interview]
Thank you very much for your honesty and willingness to participate in this discussion. I have learned a lot from talking with you, and our study team really appreciates your input.
