# Supplementary material for: Feasibility and Acceptability of a Positive Psychological Intervention for Patients With Metastatic Breast Cancer: Pre-Post Pilot Study
Source: JMIR Form Res. 2025 Oct 7;9:e77636. doi: 10.2196/77636 (PMC12504039; doi:10.2196/77636)
Supplement: Multimedia Appendix 2 [file formative-v9-e77636-s002.pdf]

TRANSCRIPT SUMMARY

Prepared by:
Participant ID:
Interview date:

**Experiences in the Study**
•   •   •
**Intervention Schedule**
•   •   •
**Positive Emotion Skills**
•   •   •
**Alternative Study Designs**
•   •   •
**Additional Input**
•   •   •
**Key Quotes**
•   •   •   •
**Other**
•   •   •
